# Effects of protein conformational transition accompanied with crosslinking density cues in silk fibroin hydrogels on the proliferation and chondrogenesis of encapsulated stem cells

**DOI:** 10.1093/rb/rbaf019

**Published:** 2025-03-20

**Authors:** Guolong Cai, Weikun Zhao, Tianhao Zhu, Ana L Oliveira, Xiang Yao, Yaopeng Zhang

**Affiliations:** State Key Laboratory of Advanced Fiber Materials, Shanghai Engineering Research Center of Nano-Biomaterials and Regenerative Medicine, College of Materials Science and Engineering, Donghua University, Shanghai 201620, People’s Republic of China; State Key Laboratory of Advanced Fiber Materials, Shanghai Engineering Research Center of Nano-Biomaterials and Regenerative Medicine, College of Materials Science and Engineering, Donghua University, Shanghai 201620, People’s Republic of China; State Key Laboratory of Advanced Fiber Materials, Shanghai Engineering Research Center of Nano-Biomaterials and Regenerative Medicine, College of Materials Science and Engineering, Donghua University, Shanghai 201620, People’s Republic of China; Universidade Católica Portuguesa, CBQF—Centro de Biotecnologia e Química Fina—Laboratório Associado, Escola Superior de Biotecnologia, Porto 4169-005, Portugal; State Key Laboratory of Advanced Fiber Materials, Shanghai Engineering Research Center of Nano-Biomaterials and Regenerative Medicine, College of Materials Science and Engineering, Donghua University, Shanghai 201620, People’s Republic of China; State Key Laboratory of Advanced Fiber Materials, Shanghai Engineering Research Center of Nano-Biomaterials and Regenerative Medicine, College of Materials Science and Engineering, Donghua University, Shanghai 201620, People’s Republic of China

**Keywords:** silk fibroin hydrogel, dynamic material microenvironment, conformational transition, 3D cell culture, cell behavior regulation

## Abstract

Silk fibroin (SF) hydrogels possess excellent biocompatibility and biomimetic properties of the extracellular matrix. Among them, the mild chemical crosslinked SF hydrogels show great application potential in the fields of 3D cell culture and tissue repairing and thus have attracted widespread attention. However, the mobility of hydrophobic chain segments of SF molecules in these chemical crosslinked hydrogels can easily cause the molecules to undergo a self-assembly process from random coil to *β*-sheet conformation due to its lower energy state, thus inducing an inevitable conformational transition process. This process further leads to dynamic changes of important material features, such as the hydrogel pore size and mechanical properties, which can probably bring some non-negligible and unknown impacts on cell behaviors and their biomedical applications. In this study, a typical mild crosslinking system composed of horseradish peroxidase and hydrogen peroxide was chosen to prepare SF hydrogels. A feasible protein conformational transition rate controlling strategy based on hydrogel crosslinking density regulation was also proposed. Our results demonstrate that the lower the hydrogel crosslinking density, the faster the conformational transition rate. Subsequently, SF hydrogels with different conformational transition rates were successfully constructed to investigate the impact of the protein conformational transition rate accompanied with initial crosslinking density on the proliferation and chondrogenic differentiation of encapsulated stem cells. Results comprehensively illustrated that the conformational transition process could effectively regulate cell behavior. The hydrogel with an appropriate conformational transition rate obviously promoted the proliferation and chondrogenesis of encapsulated stem cells, while too fast or too slow transition processes slowed down these cell activities. These findings are hopefully to provide valuable guidance for the development and efficient usage of SF hydrogels in the fields of 3D cell culture and tissue engineering.

## Graphical abstract

**Figure:**
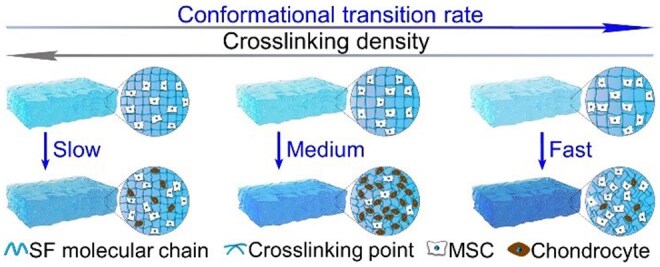

## Introduction

As a typical natural protein material, silk fibroin (SF) owns numerous advantages, such as good biocompatibility, excellent mechanical properties, adjustable biodegradability and easiness to obtain and process, thus making it widely studied and developed in the field of biomaterials [1–7]. SF can be processed into various forms of biomaterials, including microspheres [8], fibers [9], films [10], electrospun scaffolds [11–14], hydrogels [15, 16], 3D printing and cast scaffolds [17–19]. Among them, SF hydrogels (SFHs) can absorb a large amount of water while maintaining structural integrity and exhibit excellent extracellular matrix (ECM)-mimicking properties [16, 20, 21], making them highly promising for biomedical applications.

Based on the crosslinking principle, hydrogels can be divided into physical crosslinked and chemical crosslinked hydrogels. Physical crosslinked SFHs are usually formed by stimuli such as ultrasound, shear oscillation and surfactants [22–24]. These hydrogels present mild gelation conditions, but longer gelation time and relatively weaker mechanical properties, which limit their biomedical application ranges at some extent. In contrast, SFHs formed by mild chemical crosslinking strategies possess comprehensive advantages including mild gelation condition, short gelation time, better ECM-mimicking features and a wide range of adjustable mechanical properties, thus holding greater application potentials in the fields of drug sustained releasing [25, 26], wound dressing [27, 28], live cell encapsulation [29, 30] and tissue engineering [17, 31–35]. Therefore, the design and development of SFHs with effective cell behavior-regulation property based on mild chemical crosslinking strategies has become a hot research topic in recent years.

Until now, some frontier studies have confirmed that typical mild chemical crosslinked SFHs could be effectively used for 3D cell culture, *in vitro* tissue-engineered cartilage construction and *in vivo* cartilage and other tissue repairing [19, 29, 36, 37]. However, the free movement and self-assembly of the hydrophobic chain segments of SF molecules in these hydrogels will cause the proteins to transit from random coil to *β*-sheet conformation with a lower energy state, leading to an inevitable dynamic conformational transition of SF within these hydrogels (as schematically illustrated in Supplementary Figure S1) [38, 39]. These transitions can dynamically alter the basic characteristics of the hydrogels, such as the pore size, modulus and transparency. Classical research on cell–material interactions has proved that the physical and chemical features of materials, such as the pore size [40, 41], porosity [5, 40], stiffness [42, 43], topological structure [5, 14, 44–49] and surface chemistry [50–53] can all profoundly affect cell behavior. In addition, some frontier studies have also reported that the dynamical mechanical stimulation (hydrostatic pressure) [19] and dynamic change of material features, such as the material degradation process [54, 55] and the dynamic viscoelastic characteristics of materials constructed by dynamic chemical bonds [56–60] could also significantly regulate cell function. In addition, the dynamic ultrasonic and fluid shear stress stimulations could also have important impacts on cell behaviors [61]. Based on this, the microenvironmental changes of protein conformational transition within the aforementioned SFHs are likely to have an important regulatory effect on cell behavior, thus bringing a category of non-negligible and unknown impacts to the application of such an important biomaterial for cell encapsulation and tissue engineering. To date, no study has systematically investigated the SF conformational transition microenvironment and provide the corresponding regulation and utilization guidance.

**Figure 1.**
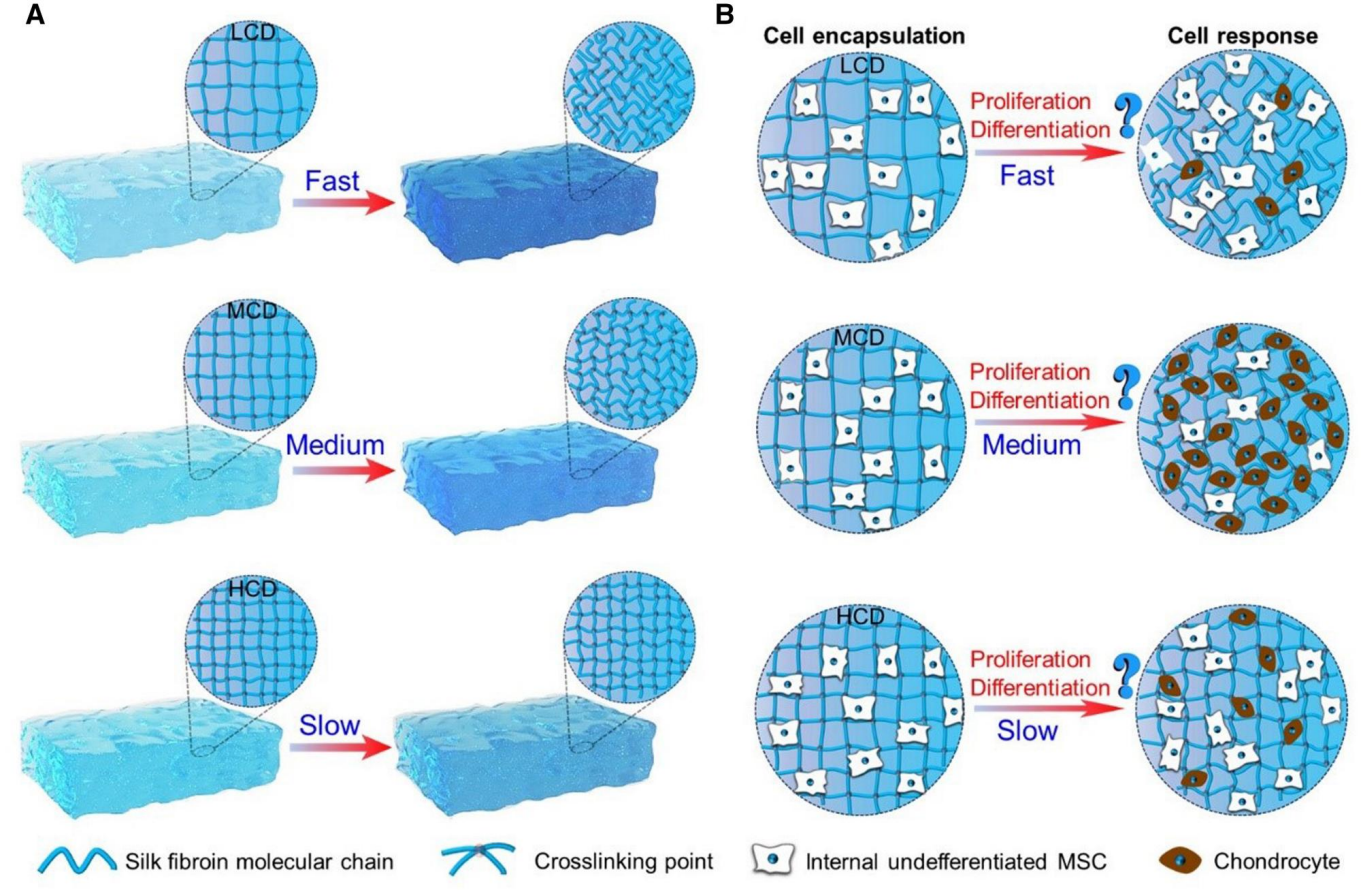
(**A**) Schematic illustration of the controlling of protein conformational transition rates in SF hydrogels through the regulation of hydrogel crosslinking density. (**B**) Schematic illustration to show the possible effects of the corresponding protein conformational transition accompanied with initial crosslinking density cues on the proliferation and differentiation of encapsulated stem cells. LCD, low crosslinking density; MCD, medium crosslinking density; HCD, high crosslinking density.

In this study, the mild chemical crosslinking strategy induced by horseradish peroxidase (HRP) was selected as the synthesis method of SFHs [62, 63]. Then, a strategy of crosslinking density modification was further developed to achieve a simple and effective regulation of the protein conformational transition rate in the corresponding SFHs. By altering the crosslinking density of SFH, the length of the freely mobile molecular chain segments of SF could be regulated. The longer freely mobile molecular chain segment (low crosslinking density) would induce faster conformation transition of the SF, and *vice versa*, thereby effectively regulating the rate of protein conformational transition within the hydrogels, as schematically illustrated in Figure 1A. Based on this strategy, a SFH material platform with different protein conformational transition rates was constructed. Focusing on the application scene of SFH for cartilage tissue repair, this study further investigated the effects of protein conformational transition rate accompanied with initial crosslinking density cues on the proliferation and chondrogenic differentiation of encapsulated bone marrow mesenchymal stem cells (MSCs), as schematically shown in Figure 1B. This work is expected to address the gap in understanding the impact of protein conformational transition rates in SFHs on stem cell behavior, offering new insights and guidance for the effective development and application of SFHs and other similar protein-based biomaterials in cartilage tissue engineering.

## Materials and methods

### Preparation of SF lyophilized powder

The detailed process parameters could be found in our previous reports [64, 65]. Briefly, the pretreated silkworm cocoons (Guizhou New Silk Road & Beautiful Life Technology Co., Ltd) are boiled in proper Na_2_CO_3_ aqueous solution (analytical pure, Sinopharm Chemical Reagent Co., Ltd) to remove sericin. Subsequently, the degummed silk is dissolved in an appropriate lithium bromide aqueous solution (analytical pure, Lithium Industrial Co., Ltd, Shanghai) and then subjected to centrifugation, filtration and dialysis by Cellulose dialysis bag (14 000 ± 2000 MWCD, Yuanju Biotechnology Co., Ltd, Shanghai) to obtain a low-concentration SF aqueous solution. After that, the solution is concentrated by cool air flow (4–8°C) to obtain an ∼10 wt% SF aqueous solution, which is then lyophilized to produce SF lyophilized powder for further application.

### Preparation of SFHs with different crosslinking densities

Based on the existing reports [66], this study sets the optimal ratio of HRP (Type VI, Sigma-Aldrich) to H_2_O_2_ (30%, Sinopharm Chemical Reagent Co., Ltd) as 50 U/ml: 12.9 mM (final concentration in the reaction system) for the preparing of SFHs. By fixing the amount of SF while varying the HRP/H_2_O_2_ dosage proportionally, SFHs with different crosslinking densities are prepared. The specific formulations of the hydrogel precursor solutions could be found in Supplementary Table S1. The prepared hydrogels were named as SFH-1, SFH-2, SFH-3, SFH-4 and SFH-5, sequentially from high to low HRP/H_2_O_2_ dosage, respectively. More specific preparation processes are as follows: SF powder, HRP powder and 30 wt% H_2_O_2_ solution were added to phosphate buffered saline (PBS) solution to obtain a 5 wt% SF solution, a 1000 U/ml HRP solution and a 490 mM H_2_O_2_ solution, respectively. Then, according to the formulations shown in Supplementary Table S1, the required SF solution, HRP solution and H_2_O_2_ solution were mixed evenly and incubated at a constant temperature (37°C) environment for 0.5 h to form the corresponding SFHs.

### Covalent crosslinking densities characterization of the fabricated SFHs

According to the previously reported methods [67], the fluorescence emission intensity of di-tyrosine bonds in the hydrogel at 410 nm after 315 nm light excitation can be used to quantitatively analyze the covalent crosslinking densities of SFH. Therefore, this study used a steady-state/transient fluorescence spectrometer (Proteostasis Therapeutics, Inc., type QM/TM) to evaluate the covalent crosslinking densities of SFHs prepared in Section ‘Preparation of SFHs with different crosslinking densities’. The specific steps are as follows: all SFHs are prepared into cylindrical shapes with a diameter of 14 mm and a height of 5 mm, then the related emission fluorescence spectrums of the corresponding hydrogels are tested using the fluorescence spectrometer. After that, the fluorescence emission intensity at 410 nm in the spectrum was statistically analyzed to reflect the relative covalent crosslinking densities of the corresponding hydrogels.

### Evaluation of the conformational transition processes of different SFHs and their impact on hydrogel features

The fabricated SFHs (SFH-1 to SFH-5) were incubated in a simulated cell culture environment (immersed in PBS, 37°C), with fresh PBS (HyClone) replaced every 2 days and a total incubation period of 14 days (unless otherwise indicated). During the incubation process, samples were taken out at indicated time points to characterize and compare the *β*-sheet conformation contents, light transmittance and macroscopic transparency state. Ultimately, the rates of conformational transition of different SFHs were comprehensively assessed by monitoring the change rates of these characteristic parameters. In addition, the micromorphology and compressive mechanical properties of the hydrogels at the corresponding incubation time points were also characterized to further reveal the impacts of the protein conformational transition rate on the micromorphology and mechanical properties of the SFHs.

#### Characterization of the β-sheet conformation content changes of different SFHs during the incubation process

After 1, 4, 7, 10 and 14 days of incubation, the hydrogel materials were taken out and subjected to lyophilization. Subsequently, the materials after freeze-drying were tested using a Fourier transform infrared (FT-IR) spectrometer (Thermo Fisher, Nicolette 6700) with an attenuated total reflection accessory, scanning over a wavelength range from 400 to 4000 cm^−1^ with a resolution of 4 cm^−1^. The FT-IR spectral data in the amide I region, between 1600 and 1700 cm^−1^, were selected from the corresponding spectral plots. The peaks were deconvoluted using second-derivative fitting with Peak-fit software, and the content of the *β*-sheet conformation in the related SFH was calculated. After peak fitting, the content of the *β*-sheet conformation was determined as the ratio of the peak area within the range of 1616–1637 cm^−1^ to the total peak area within the range of 1600–1700 cm^−1^ according to the previous report [63].

#### Investigation of the light transmittance changes of different SFHs during the incubation process

After 1, 3, 5, 7, 9, 11, 13 and 15 days of incubation, use a microplate reader (Thermo Fisher, Type Multiskan FC) to measure the optical density (OD) value of the SFHs at 620 nm. The OD value changes were used to quantitatively reflect the changes in light transmittance of the SFHs, thus indirectly reflecting the conformational transition rates of the hydrogels. The OD value change ratio of the corresponding hydrogel at the indicated incubation time point was calculated using equation (1):


(1) OD value change ratio = (ODt-OD0)OD0×100%#

where *OD_0_* represents the initial OD value of the hydrogel, and *OD_t_* represents the OD value measured after *t* days of incubation.

#### Observation of the macroscopic transparency state changes of different SFHs during the incubation process

After incubating the hydrogel samples in a simulated cell culture environment for 1 day and 10 days, remove them from the 12-well plate and place them on a transparent substrate marked with ‘SFH’. Then, photograph the hydrogels from above to qualitatively record the changes in their transparency.

#### Characterization of the cross-sectional micromorphology features of different SFHs during the incubation process

The cross-sectional micromorphology features of the hydrogels were characterized using a scanning electron microscope (SEM, Hitachi, FlexSEM 1000II), which could be powerfully used to evaluate the pore size and other internal microstructure characteristics. The specific process was as follows: 1 ml of the hydrogel precursor solution was added per well to a 24-well plate to prepare the hydrogels. After gelation, the hydrogels of SFH-1 to SFH-5 were incubated in a simulated cell culture environment. After incubation for 1, 4, 7, 10 and 14 days, the hydrogel samples were taken out. Then, the samples were longitudinally cut open and subjected to freeze-drying treatment. After that, the section surface was then placed facing up on a sample stage coated with conductive adhesive and then gold-sputtered for 90 s (at a current of 10 mA). Finally, the cross-sectional micromorphology features were photographed using SEM at a voltage of 1 kV.

#### Investigation of the compressive mechanical properties of different SFHs during the incubation process

The compressive mechanical properties of different SFHs after 1, 4, 7, 10 and 14 days of incubation were tested using an electronic universal testing machine (Hengyu Instrument, HY-941). All the tested samples were cylindrical shape with a diameter of 14 mm and a height of 10 mm. During the testing process, the compression rate is set as 10 mm/min. The compressive modulus and compressive strength were calculated from the stress–strain curve according to the previous reports [18, 38]. Specifically, the compressive modulus is obtained by fitting the slope of the straight line within the strain range from 5 to 15% on the corresponding stress–strain curves. The compressive strength is the stress value at a strain of 30% on the related stress–strain curves.

### Investigation of the proliferation and differentiation of MSCs encapsulated within the different SFHs

In the cell experiments, the cell culture medium was prepared by mixing DMEM medium (Gibco), fetal bovine serum (Gibco) and penicillin–streptomycin (Gibco) in a volume ratio of 100:10:1. In the assessment of cell proliferation, the DMEM medium used is low-glucose DMEM medium. While for the cell differentiation assessment experiment, it is entirely replaced with high-glucose DMEM medium.

#### Encapsulation and cultivation of MSCs within the different hydrogels

SF lyophilized powder, HRP powder and 30% H_2_O_2_ solution were added separately to the DMEM medium to obtain a concentration of 5 wt% SF solution, 1000 U/ml HRP solution and 490 mM H_2_O_2_ solutions. The solutions were then sterilized by filtering through sterile filters (0.22 μm) separately. Subsequently, a suspension of MSCs (passages 3–5) was prepared. The corresponding cell density of the cell suspension was counted and calculated using a hemocytometer.

Then, the corresponding volume of cell suspension (calculate based on the total cell numbers required) was aspirated into a new centrifuge tube and centrifuged (1000 rpm, 3 min) to remove the supernatant. The sterilized SF solution, HRP solution and H_2_O_2_ solution were mixed evenly to form the precursor solution of the corresponding hydrogels according to Supplementary Table S1. The required volume of hydrogel precursor solution was aspirated and quickly mixed with the cells at the bottom of the aforementioned centrifuge tube, and the cell density in the hydrogel precursor solution was 8 × 10^5^ cells/ml. Then the stem cell containing hydrogel precursor solution was added to a 24-well plate; the volume per well was 500 μl. After that, the 24-well plates were placed in a 37°C cell culture incubator (5% CO_2_) to form corresponding cell-laden hydrogels. After gelation (∼0.5 h incubation), the cell-laden SFHs in the wells were washed three times with fresh cell culture medium (30 min per time) to remove any residual small molecules from the hydrogels. Subsequently, the encapsulated MSCs were cultured in the 37°C cell culture incubator (5% CO_2_), and fresh cell culture medium was changed every 2 days.

#### Assessment of the proliferation of MSCs encapsulated in the different SFHs

As for the cell viability test of 3D cell culture system, the permeability of cell viability test assay is quite important. Compared with the commonly used Cell Counting Kit-8 assay for 2D cell culture system, the Cell Titer-Glo 3D Cell Viability Assay (CTG, Promega) could more easily penetrate into the hydrogel to react with cells, thus more suitable for the viability evaluation of cells encapsulated in the hydrogels [39, 68]. The CTG assay was used to evaluate the total cell viability after 1, 4 and 7 days of cultivation, which could be used for reflecting the relative cell proliferation status [39]. Specifically, CTG reagent was uniformly mixed with PBS at a 1:2 (v: v) ratio and then heated to 37°C using a water bath to obtain the CTG working solution. Subsequently, after the indicated culture time, the cell culture medium in the 24-well plates was aspirated and replaced by the CTG working solution (500 μl per well) and then placed on a shaker (shaker speed 300 rpm) at room temperature under dark conditions for 1 h to allow the CTG working solution to permeate the hydrogel and react completely with the intracellular adenosine triphosphate. Finally, the reacted CTG working solution was transferred to a 96-well plate (100 μl per well), and its luminescence units (LU) were measured using the ‘Luminescence’ testing mode of a multifunctional microplate reader (Tecan, Infinite 200).

#### Live/dead staining of MSCs encapsulated in the typical SFHs

In order to more directly observe the total cell numbers and live/dead status in the 3D culture environment, typical hydrogel groups (SFH-1, SFH-3, SFH-5) were selected for live/dead staining after 7 days of culture, followed by the observation and capture of relevant fluorescent images using confocal microscopy. More specifically, an appropriate amount of 10× Assay Buffer solution (Shanghai Yeason) was taken and diluted 10 times with deionized water to obtain 1× Assay Buffer. Subsequently, Calcein-AM solution (2 mM, Shanghai Yeason), PI solution (1.5 mM, Shanghai Yeason) and 1× Assay Buffer were uniformly mixed at a ratio of 1:3:1000 (v:v:v) to prepare the staining working solution. And the final concentrations of Calcein-AM and propidium iodide (PI) in the working solution were 2 μM and 4.5 μM, respectively. After that, the cell culture medium in the 24-well plates was aspirated, and the hydrogels were rinsed twice with 1× Assay Buffer (each time for 10 min). Then, the staining working solution was added (300 μl per well), and the plate was incubated in a cell culture incubator for 1 h. After incubation, the staining working solution was aspirated, and the samples were rinsed twice with 1× Assay Buffer (each time for 10 min) to remove any residual staining reagent. Subsequently, a laser scanning confocal microscopy (Zeiss, LSM700) was used to observe and capture the corresponding fluorescent images under excitation wavelengths of 488 and 561 nm. Typical regions were also selected for Z-axis scanning observation.

#### Assessment of the chondrogenic differentiation abilities of MSCs encapsulated in the different SFHs

The chondrogenic differentiation levels of MSCs encapsulated in SFHs were assessed by detecting the expression of characteristic chondrogenic genes after 7 and 14 days of culture, respectively. The selected chondrogenic-specific genes contain Col II (Type II Collagen), ACAN (Aggrecan) and PRG4 (Proteoglycan 4, also known as lubricin), with GAPDH as the reference gene.

The relevant detection experiments were conducted by Shanghai Dai Xuan Biotechnology Co., Ltd after 7 or 14 days of cell culture in the high-glucose DMEM medium, the indicated hydrogels were quickly transferred from the well plate to the pre-cooled mortar for grinding and crushing until the hydrogel became powder. Before sample transfer, an adequate amount of liquid nitrogen was added to a mortar to pre-cool it thoroughly. During the grinding and crushing process, the liquid nitrogen was continuously added to the mortar to keep it cool. Next, the powdered hydrogel–cell composites were quickly transferred to proper centrifuge tubes; TRI-zol solution (Shanghai Yuanye), at 1 ml per tube, was added, and then the mixture was vortexed for lysis. The tubes were then placed at room temperature for 10 min before being transferred to a −80°C refrigerator for storage. Finally, the corresponding RNA extraction and real-time quantitative polymerase chain reaction (RT-PCR) analysis were carried out as previous reports [18, 19]. The primer information for the related genes in RT-PCR analysis is provided in Supplementary Table S2. Data were analyzed using the 2^−ΔΔCt^ strategy to calculate the relative gene expressions of Col II, ACAN and PRG4 among SFH-1–SFH-5.

### Statistical analysis

In order to enhance the accuracy and reliability of the experiment results, the number of parallel samples is no less than 3. The experimental results are presented as the mean value ± standard deviation. Data differences were statistically analyzed using one-way ANOVA in origin software. A probability value of *P *<* *0.05 indicates that the experimental results have significant statistical differences.

## Results and discussion

### Construction of SFHs with different conformational transition rates

By altering the crosslinking density of SFH, the length of the freely mobile molecular chain segment of SF within the hydrogel could be regulated. Based on the fundamental theory of polymer physics, it can be inferred that the longer, freely mobile molecular chain segments (low crosslinking density) would induce faster conformational transition of the SF, and *vice versa*. Based on this, we have proposed a strategy to fabricate the SFHs with different protein conformational transition rates. Specifically, this study constructed different SFHs (SFH-1 to SFH-5) by fixing the amount of SF while changing the dosage of enzyme and the corresponding assistant reagent (HRP/H_2_O_2_).

**Figure 2.**
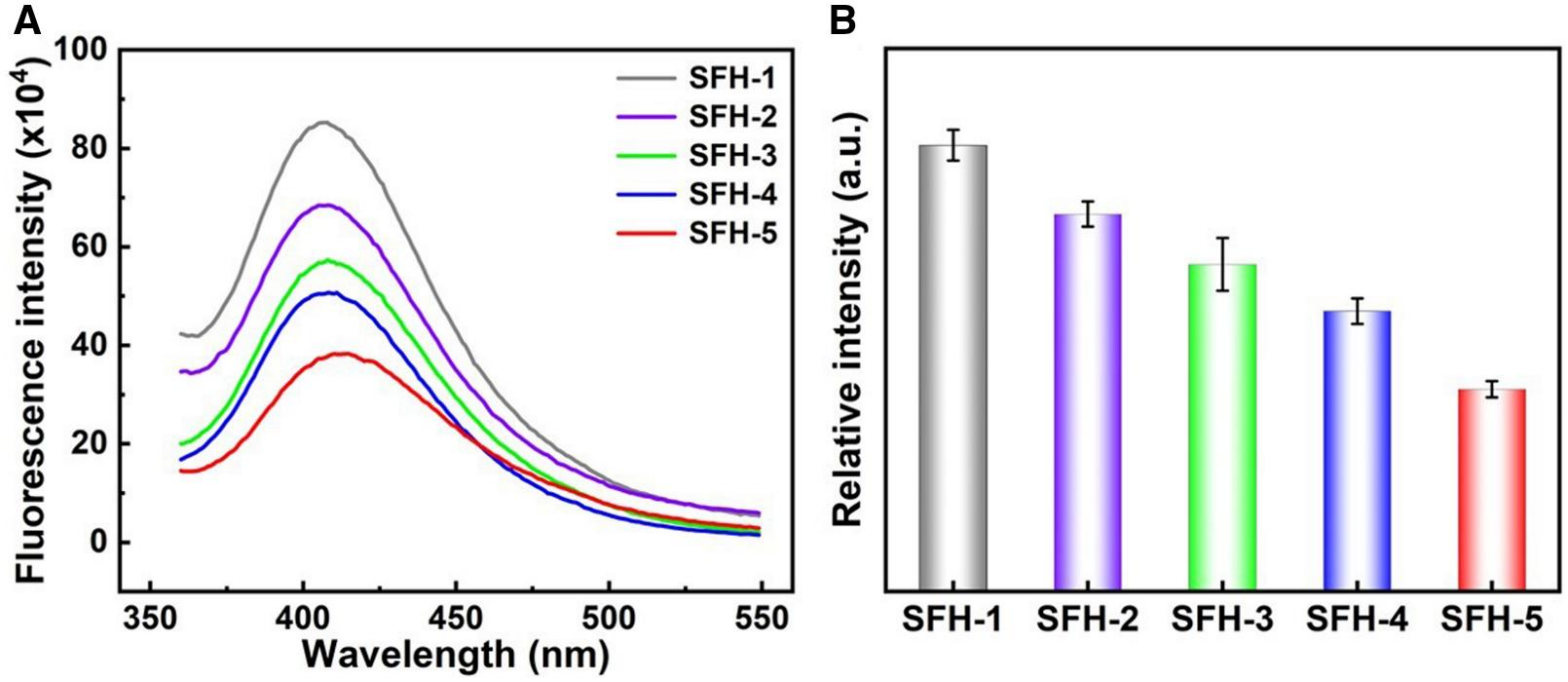
Covalent crosslinking density evaluation of the indicated SF hydrogels. (**A**) Fluorescence spectra of the indicated SFHs. (**B**) The relative fluorescence intensity at 410 nm of the indicated SFHs.

In the SFH formed by the HRP-catalyzed crosslinking system, SF molecules are interconnected through di-tyrosine bonds to form the corresponding covalent crosslinking network [4, 62]. Therefore, the amount of di-tyrosine bonds in the SFH could be used to quantitatively evaluate the covalent crosslinking density of the hydrogel. Figure 2A shows the fluorescence spectra of the fabricated hydrogels of SFH-1 to SFH-5, and the relative fluorescence intensity at 410 nm could be effectively used for estimating the amount of di-tyrosine bonds accordingly [63, 69]. The relative emission fluorescence intensity at 410 nm of the indicated hydrogels is further shown in Figure 2B, which directly showed that the fluorescence intensity was gradually decreased from SFH-1 to SFH-5. It has been reported that higher fluorescence intensity at 410 nm reflect higher amount of di-tyrosine bonds and covalent crosslinking density [63]. Consequently, as the amount of HRP/H_2_O_2_ decreases (from SFH-1 to SFH-5), the covalent crosslinking density of the hydrogel gradually decreased. In summary, fixing the amount of SF while changing the amount of HRP/H_2_O_2_ could feasibly and effectively regulate the covalent crosslinking density of SFHs. The order of hydrogel crosslinking density from high to low is SFH-1, SFH-2, SFH-3, SFH-4 and SFH-5.

Then, this research incubated the hydrogels in a simulated cell culture environment (PBS, 37°C) and evaluated their conformational transition processes through the *β*-sheet conformation content characterization and the hydrogel transmittance measurement during the incubation process of corresponding hydrogels (SFH-1–SFH-5). The conformational transition of SF molecules in the hydrogels involves a transition from random coil conformation to a lower energy state of *β*-sheet conformation, and the increasing of *β*-sheet conformation content decreases the transmittance of the hydrogels.

**Figure 3.**
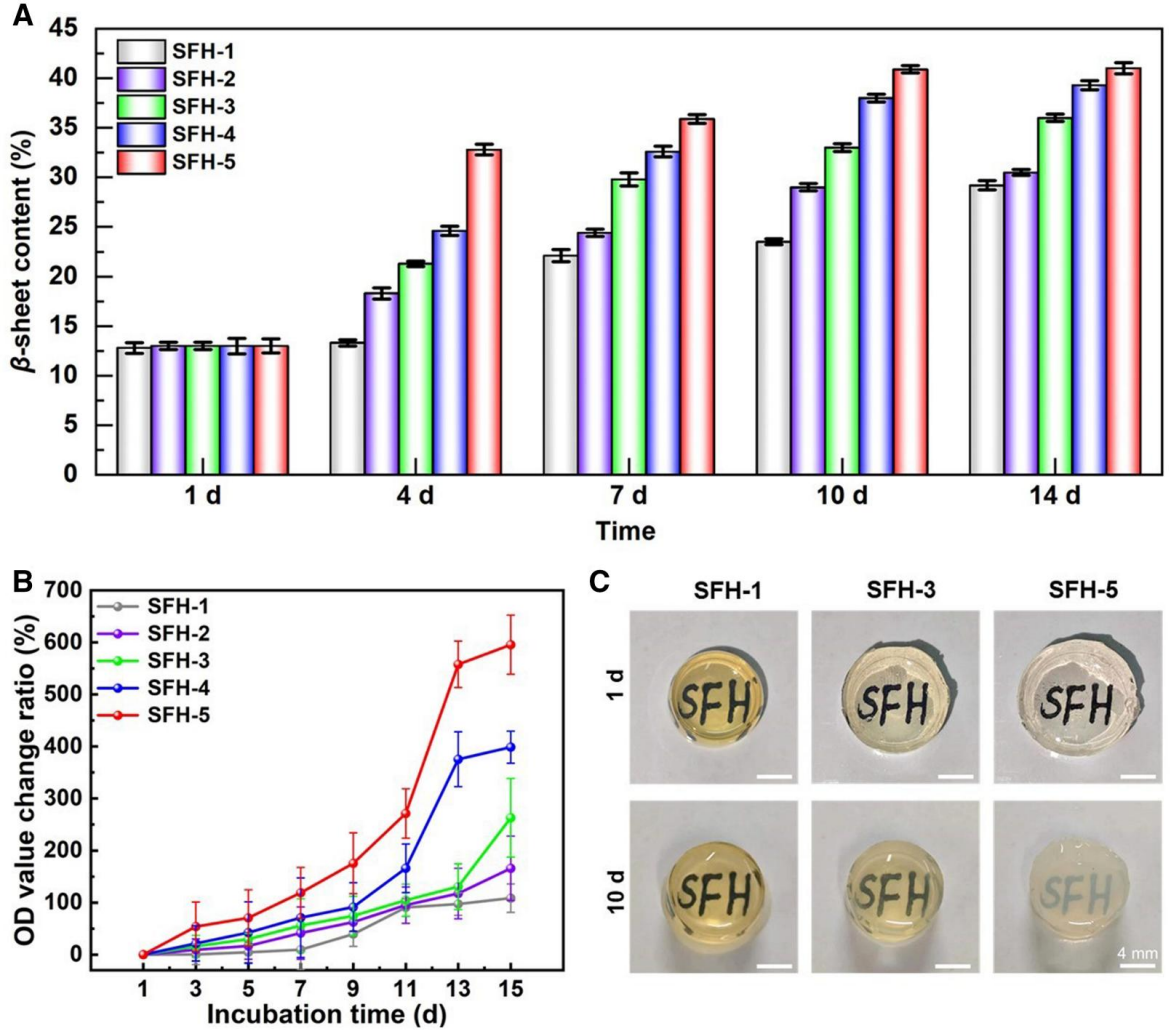
Analysis of the conformational transition process of the SF hydrogels after indicated incubation times in the simulated cell culture environment. (**A**) *β*-sheet content of the proteins in the hydrogels, (**B**) absorbance change ratio of the hydrogels at 620 nm and (**C**) gross views of the corresponding typical hydrogels.

FTIR is an effective strategy to characterize the *β*-sheet conformation content in SF and other protein materials [70, 71]. After 1, 4, 7, 10 and 14 days of incubation in the simulated cell culture environment, the FTIR spectra from 2500 to 1250 cm^−1^ of the SFHs are shown in Supplementary Figure S2. The results show that at day 1, the characteristic peaks of each hydrogel group appeared at 1655–1645 cm^−1^, which belong to the characteristic peaks of the random coil conformation of SF in the amide I region [63], indicating that the main initial protein conformation in all the hydrogels was random coil conformation. As the incubation time gradually increased, a strong absorption peak ∼1635–1633 cm^−1^ (reflecting to the *β*-sheet conformation) obviously appeared in SFH-5 (10 days) and SFH-4 (14 days), which generally indicated that the conformational transition rate of SFH-5 and SFH-4 is faster than other groups. In order to more intuitively and quantitatively present the changes in the content of *β*-sheet conformation in the SFHs, this study further used Peakfit software to perform peak deconvolution on the amide I region of the obtained spectra (Supplementary Figure S2) to calculate the contents of *β*-sheet conformation in the hydrogels [71]. Related results are presented in Figure 3A. At the initial state (1 day of incubation), there was no significant difference among the contents of *β*-sheet conformation in different SFHs (SFH-1–SFH-5), all around 13%. However, after incubating for 4 days, the contents of *β*-sheet conformation in SFH-1 to SFH-5 increased to 13.3, 18.3, 21.3, 24.6 and 32.8%, respectively. The trends after incubating for 7, 10 and 14 days were consistent. As the incubation time increased, the contents of *β*-sheet conformation in each group were also gradually increased, and the conformational transition rate gradually increased from SFH-1 to SFH-5 (see Figure 3A). Overall, the content of *β*-sheet conformation in the SFH-5 group increased from ∼13.5 to ∼41% within 14 days, with an increase amount of ∼27.5%. However, the increase amount of SFH-1 to SFH-4 groups were ∼16.4, 17.5, 23.0 and 26.1%, respectively. In addition, it can also be seen that the content of *β*-sheet conformation in the SFH-5 group is almost unchanged from 10 to 14 days of incubation (both around 41%); that is probably because the fastest conformational transition rate of this group made it change to the maximum *β*-sheet conformation content after 10 days of incubation. These results have directly revealed that the conformational transition rate from slow to fast is in the order of SFH-1, SFH-2, SFH-3, SFH-4 and SFH-5, showing an inverse correlation with the hydrogel crosslinking density.

In addition, this study also characterized the absorbance of different hydrogels at indicated incubation times to reflect the transmittance changes and related conformational transformation process. The calculated OD value change ratios are shown in Figure 3B. Comprehensively, the change ratio from slow to fast is in the order of SFH-1, SFH-2, SFH-3, SFH-4 and SFH-5, which is highly consistent with the increasing trend of *β*-sheet content in the SFHs (Figure 3A). For example, after 7 days of incubation, the absorbance of SFH-1 to SFH-5 increased to 9.5, 41.4, 58.0, 70.9 and 119.0%, respectively, and the value changed to 97.3, 117.4, 130.7, 375.6 and 557.9% after 13 days of incubation. Moreover, the gross view of the typical hydrogels (SFH-1, SFH-3, SFH-5) after incubation for 1 and 10 days was also captured (see Figure 3C) to show the transparency changes more intuitively. The initial gross appearance showed that SFH-1 is darker than other groups, and the initial diameter size of SFH-1 is smaller than other groups. As for the different initial color, it is mainly due to the fact that the amount of HRP (slightly yellow colored) used in SFH-1 is higher than other groups. As for the different initial size, it is probably because the covalent crosslinking density of SFH-1 is higher than other groups (Figure 2B), thus leading to a smallest volume expansion after water absorption. Moreover, as the conformational transition occurs, the transparency change rate of SFH-5 is significantly faster than other groups, and the change rate of SFH-3 is between SFH-1 and SFH-5. The gross view appearance of the five SFHs and their change laws (see Supplementary Figure S3) all follow the above rules obtained from the typical three groups (Figure 3C). Overall, as the incubation time increased, the trend of the optical transparency to decrease is consistent with the trend of *β*-sheet conformation content to increase. This is attributed to the increase of *β*-sheet conformation content in the SFH, which effectively reduces its optical transparency [38, 39].

In summary, the changes of *β*-sheet conformation content and optical transparency of the corresponding hydrogels both confirmed that the fabricated hydrogels have different conformational transition rates. Additionally, the conformational transition rate of the proteins in the SFH generally shows an inverse correlation with the hydrogel crosslinking density.

### Impact of the different protein conformational transition processes on the micromorphology features of SFHs

**Figure 4.**
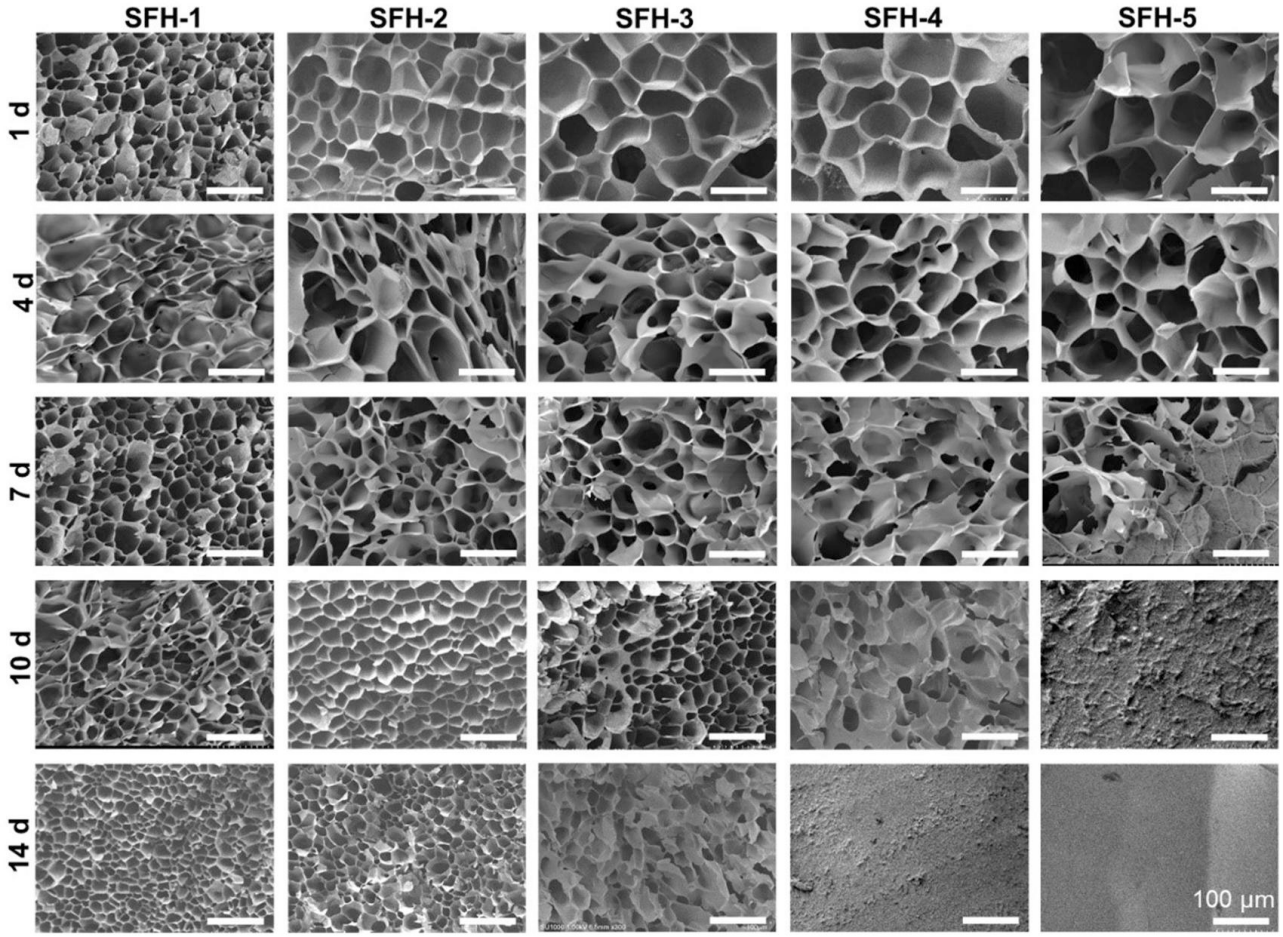
SEM images of the section of the SF hydrogels after indicated incubation times in the simulated cell culture environment.

Protein conformational transition in SFHs could probably change the hydrogel’s micromorphology features and other related characteristics. In order to reveal these impacts, this study used SEM to systematically characterize the cross-sectional micromorphology feature changes of the indicated hydrogels during the protein conformational transition process. Results illustrated that the five kinds of SFHs all exhibited obvious porous structures at the initial stage (after 1 day of incubation), and the pore size gradually increased from SFH-1 to SFH-5, as shown in the first row of Figure 4. This is related to the gradual decrease in crosslinking density from SFH-1 to SFH-5 (Figure 2B). Relevant literature has also reported an increase in pore size of hyaluronic acid tyramine hydrogels with the decrease in crosslinking density [72].

After 4–7 days of incubation, the pore sizes of SFH-5 and SFH-4 sharply decreased, while those of SFH-3, SFH-2 and SFH-1 decreased more gradually. After 10–14 days of incubation, the pore sizes of SFH-5 and SFH-4 further declined rapidly, and distinct pore structures were difficult to observe under the sample preparation and observation conditions used in this study (Figure 4). During this period, the pore sizes of SFH-3 and SFH-2 also sharply decreased, whereas the reduction in SFH-1 was relatively slight (Figure 4).

The amplitude and rate of decreasing in hydrogel pore size were both positively correlated with the conformational transition rate, which showed an order of SFH-1 to SFH-5, from slow to fast. The changes in the pore size within SFHs are likely the result of the aggregation of SF molecular chain segments to form dense *β*-sheet structures from loose random coil structures, causing the hydrogel network structure to become denser.

### Impact of the different protein conformational transition processes on the mechanical properties of SFHs

The protein conformational transition from random coil to *β*-sheet in SFHs could lead to a denser network structure, thereby dynamically changing the mechanical properties of the hydrogels. To clarify this specific impact, this study further characterized the compressive mechanical properties of the hydrogels during the incubation process in the simulated cell culture environment. The compressive stress–strain curves of the SFHs after incubation for different times are shown in Supplementary Figure S4. It can be seen that as the incubation time increases, the stress of each kind of hydrogel under the same strain were all gradually increased, indicating that the hydrogels gradually become harder. In addition, it can be also roughly obtained that the increasing amplitudes of SFH-3 to SFH-5 were significantly higher than that of SFH-1 and SFH-2 (Supplementary Figure S4).

**Figure 5.**
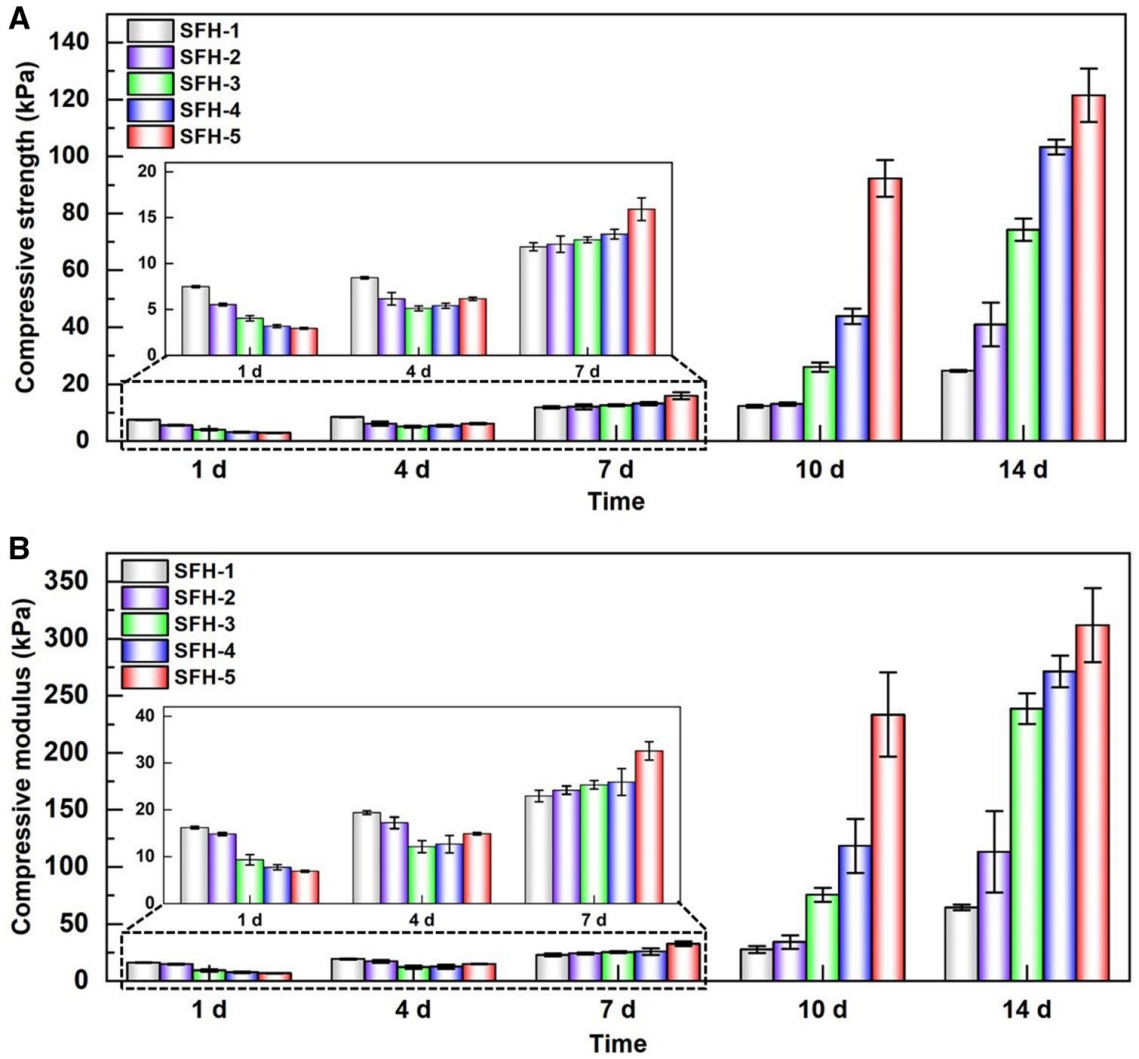
Changes in compressive mechanical properties of the SF hydrogels after indicated incubation times in the simulated cell culture environment. (**A**) Compressive strength and (**B**) compressive modulus.

In order to quantitatively compare and illustrate the impact of the protein conformational transition process on the compressive mechanical properties of SFHs, this study further calculated the compressive strength and modulus of the corresponding hydrogels based on the relevant stress–strain curves (Supplementary Figure S4). The changes in compressive strength and modulus of different hydrogels during the indicated protein conformation transition process are shown in Figure 5. Results showed that the average compressive strengths at the initial state (1 day of incubation) of SFH-1 to SFH-5 were 7.5, 5.5, 4.0, 3.2 and 2.9 kPa, respectively. The compressive modulus were 16.2, 14.8, 9.3, 7.7 and 6.9 kPa, respectively. The different initial mechanical properties are likely to be induced by the different crosslinking densities of the hydrogel. Hydrogels with higher crosslinking density presented higher compressive strength and modulus, while those with lower crosslinking density showed lower strength and modulus. Ding *et al.* [73] have also reported similar impacts of the crosslinking density on the compressive mechanical properties of suckerin-based hydrogels. On the fourth day of incubation, the compressive strength and modulus of each hydrogel increased to some extent. On the seventh day of incubation, there was a further increase, and the mechanical parameters of SFH-3–SFH-5 gradually became higher than those of SFH-1–SFH-2. At this time point, the compressive strengths of SFH-1 to SFH-5 increased to 11.8, 12.1, 12.6, 13.2 and 15.9 kPa, respectively (Figure 5A). Additionally, the compressive modulus increased to 22.9, 24.2, 25.3, 25.9 and 32.6 kPa, respectively (Figure 5B). After incubation for 10–14 days, all hydrogel’s compressive mechanical properties were further increased. The compressive strength and modulus of SFH-3–SFH-5 increased sharply, a change that was much rapid than those of SFH-1–SFH-2 (Figure 5). Comprehensively, the compressive strength and modulus of hydrogels were gradually increased with the protein conformational transition. And these features increasing rate from slow to fast is SFH-1 to SFH-5 (Figure 5), which is positively correlated with the protein conformational transition rates of the SFHs (Figure 3).

As the incubation time increased, the content of *β*-sheet conformation in the SFH gradually increased (Figure 3A), and the pore size of the hydrogel gradually decreased (Figure 4). That is probably why the compressive strength and modulus increased with the increase in incubation time. Specifically, the initial different crosslinking densities of hydrogels (SFH-1>SFH-2>SFH-3>SFH-4>SFH-5) have directly induced the initial compressive strength and modulus trends (SFH-1>SFH-2>SFH-3>SFH-4>SFH-5). As the protein conformational transition occurs, the compressive strength and modulus of hydrogels with fast conformational transition (SFH-4 and SFH-5) quickly increased and even exceeded those features of SFH-1 and SFH-2 after 7 days of incubation (Figure 5). As the incubation time further increased to 10 and 14 days, the mechanical properties of SFH-4 and SFH-5 were further sharply increased. The mechanical features increasing trend of SFH-3 with middle conformational transition rate is between SFH-2 and SFH-4 (Figure 5). Overall, the compressive strength and modulus changing trends were mainly determined by the initial crosslinking density and the following conformational transition rate of the SFHs. The increasing rate of the mentioned mechanical properties is positively correlated with the conformational transition rate of hydrogels. Therefore, after 7–14 days of incubation, the average compressive strength and modulus order from low to high becomes SFH-1, SFH-2, SFH-3, SFH-4 and SFH-5, which is completely opposite to the initial trends determined by the hydrogel crosslinking density.

### Effects of protein conformational transition accompanied with initial crosslinking density in SFHs on the proliferation of encapsulated MSCs

**Figure 6.**
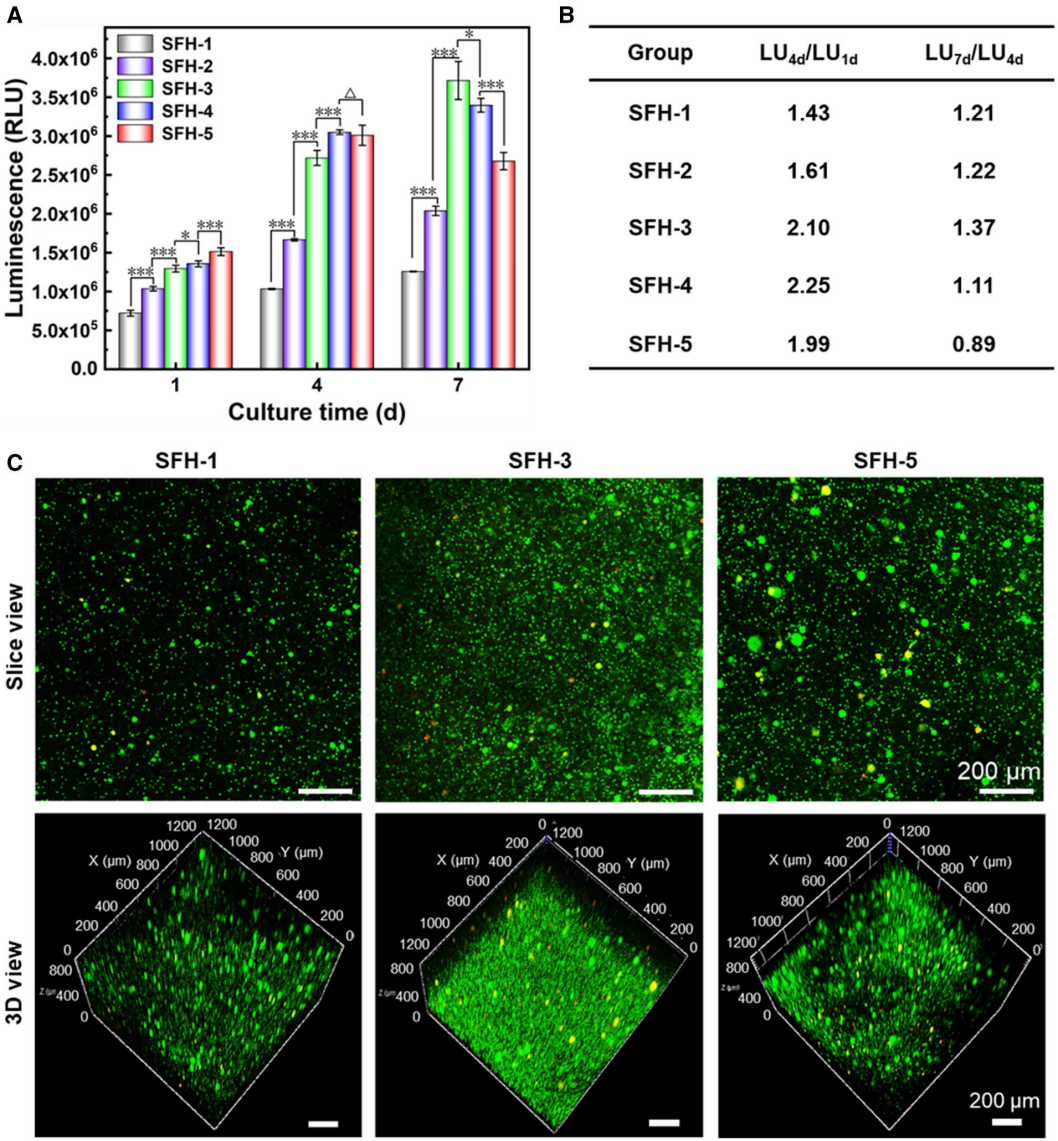
Effects of protein conformational transition rates accompanied with initial crosslinking density in SF hydrogels on the proliferation of encapsulated MSCs. (**A**) Relative cell viability changes within 7 days of cell culture, (**B**) corresponding LU value change rate of (**A**) and (**C**) laser scanning confocal microscopy images of the live/dead staining of encapsulated MSCs in the indicated SF hydrogels after 7 days of culture. All scale bars in (**C**) are 200 µm. ‘Δ’: *P *>* *0.05; ‘*’: 0.01 < *P *<* *0.05; ‘***’: *P *<* *0.001. The *P* values of the one-way ANOVA test of the data in (**A**) are listed in Supplementary Tables S3–S5.

During 7 days of culture, the relative cell viability changes were characterized by CTG reagent, which could be indirectly used to evaluate the cell proliferation of encapsulated MSCs (Figure 6A). Results showed that after 1 day of culture, a significant difference in cell viability among the groups was observed, showing a gradual increase from SFH-1 to SFH-5. After 4 days of culture, the total cell viability of all groups had significantly increased, indicating that obvious cell proliferation has occurred during this period. In order to more clearly reflect and compare the cell proliferation, corresponding LU value change rates (LU_4d_/LU_1d_) have been calculated and presented in Figure 6B. It can be found that during this culture period, the proliferation rate of cells gradually increased from SFH-1 to SFH-4, while the corresponding proliferation rate in SFH-5 is between SFH-2 and SFH-3. After 7 days of culture, the total viability of cells in the SFH-5 group decreased to some extent compared to the 4-day result, while the cell viability in other hydrogels all had a further improvement. During this process, the corresponding LU value change rate (LU_7d_/LU_4d_) has further illustrated that the cell proliferation in the SFH-3 group is the fastest, followed by SFH-2 and SFH-1, and the slowest is SFH-4 and SFH-5 (Figure 6B). It is worth noting that the cell proliferation rate in SFH-5 group is less than 1 during this culture process (4–7 days of culture), indicating that some cells inside SFH-5 may have undergone apoptosis.

To more intuitively observe the growth and survival situation of cells cultured inside the typical hydrogels, this study also recorded the live/dead staining results of the cells cultured in SFH-1, SFH-3 and SFH-5 (see Figure 6C). Results showed that after 7 days of culture, the number of the live cells (green color) in each hydrogel is far more than that of the dead cells (red color). Further comparison illustrated that the total cell number from high to low is in the order of SFH-3, SFH-5 and SFH-1, which is highly consistent with the overall cell viability trend tested by CTG reagent (Figure 6A).

As for 3D cell culture, material features such as stiffness, pore size and porosity of the substrate, as well as the dynamic mechanical stimulation in the 3D culture environment, could all significantly regulate cell proliferation. More specifically, previous reports have illustrated that substrates with larger pore size and higher porosity were more conducive to cell proliferation compared to those with smaller pore size and lower porosity in the 3D cell culture system [41], as the larger pore size and higher porosity could provide more space for cell growth and were more favorable for cell migration and nutrients transportation [74]. Recently, some dynamic mechanical stimuli and other dynamic material features have also been proved to effectively regulate the proliferation of cells cultured in 3D environment [19, 75, 76]. The SFH degradation regulated by protease enzyme has also been proved to have significant impacts on cell behaviors [77]. Although some slight SFH degradation might also happen during the 2 weeks of incubation in the present research, the degradation degree and peptide products would probably be similar with each other in all the five hydrogels. So, the material degradation effects on cell behaviors in the present study could be ignored. In this study, as the protein conformational transition occurred, the network structure of the hydrogel became denser, leading to a gradual decrease in pore size and porosity and consequently to a progressive increase in compressive strength and modulus. Comprehensively, it presented a dynamic contraction and stiffening process during the mentioned conformational transition process. And the trend and rate of the dynamical material changes were positively correlated with the rate of the conformational transition rate.

It is probable that the material features and their dynamic changes mentioned above have induced the different cell proliferation behaviors. At the initial cell culture stage (1 day), the observed trend of a gradual increase in cell viability from SFH-1 to SFH-5 is probably attributed to the gradual increase trend of initial hydrogel pore size induced by the different covalent crosslinking density (Figure 2). Specifically, SFH-5 with the lowest crosslinking density has the largest pore size, thus more beneficial for the initial cell viability maintenance and growth, and *vice versa*. During the period of 1–4 days, the pore size of the hydrogel with faster conformational transition (SFH-5) decreased obviously (Figure 4), which may have a certain slowing effect on cell proliferation. The pore size of other hydrogels only has slight changes. Combined with the difference in the initial total cell viability (SFH-1 < SFH-2 < SFH-3 < SFH-4 < SFH-5), it comprehensively led to a gradually increasing cell proliferation rate from SFH-1 to SFH-4, while the cell proliferation rate of SFH-5 was lower than SFH-3 and SFH-4. In addition, the dynamic stiffening process (changing from low to high modulus) of the hydrogel, which allows for proper conformational transitions, may also have a certain promoting effect on cell proliferation. This results in significantly higher cell proliferation in SFH-3 and SFH-4 compared to SFH-1 and SFH-2 (Figure 6B). This kind of dynamical material cue effect has been supported by Silver *et al*. [78], which shows that a proper dynamical substrate stiffening process (induced by secondary light-induced polymerization) enhances cell proliferation. During the period of 4–7 days, the conformational transition led to a further decrease in the pore size of the hydrogel, especially in SFH-5 and SFH-4 with fast conformational transition rate. Parts of the pore structures were even no longer visible for SFH-5 with the fastest conformational transition rate, and SFH-4 with the second-fastest conformational transition rate also showed a significant decrease in pore size (Figure 4). This decisive factor could lead to a significant limitation on the proliferation of cells encapsulated inside the hydrogels, thus presenting a very low rate of cell proliferation in SFH-4 and even a slight decrease in the total cell number in SFH-5 during the period of 4–7 days (Figure 6A and B). This restriction effect has also been observed in previously reported SFHs with obvious conformational transition process [79]. As for SFH-1–SFH-3 with moderate or slow conformational transition rate, a faster conformational transition still led to a faster cell proliferation rate during 4–7 days (Figure 6B). The probable reasons are listed as follows: on the one hand, the total number of cells in SFH-3 at day 4 was significantly higher than that in SFH-2, and SFH-2 was obviously more than that in SFH-1 (Figure 6A). On the other hand, the more obvious and proper dynamic stiffening process of SFH-3, compared with SFH-2 and SFH-1 (Figure 5), during this period may also have a certain promoting effect on cell proliferation.

In summary, the protein conformational transition process in SFH could significantly affect the proliferation of encapsulated cells. An appropriate dynamic conformational transition process could significantly enhance the corresponding cell proliferation, while the too slow or too fast transition process could lead to a slowdown or even restrict effects in cell proliferation during the indicated evaluating period. The aforementioned results were likely attributed to the comprehensive effects of the initial pore structures induced by different hydrogel crosslinking density and the subsequent dynamic material characteristic changes induced by the different conformational transition rates.

### Effects of protein conformational transition accompanied with initial crosslinking density in SFHs on the chondrogenesis of encapsulated MSCs

**Figure 7.**
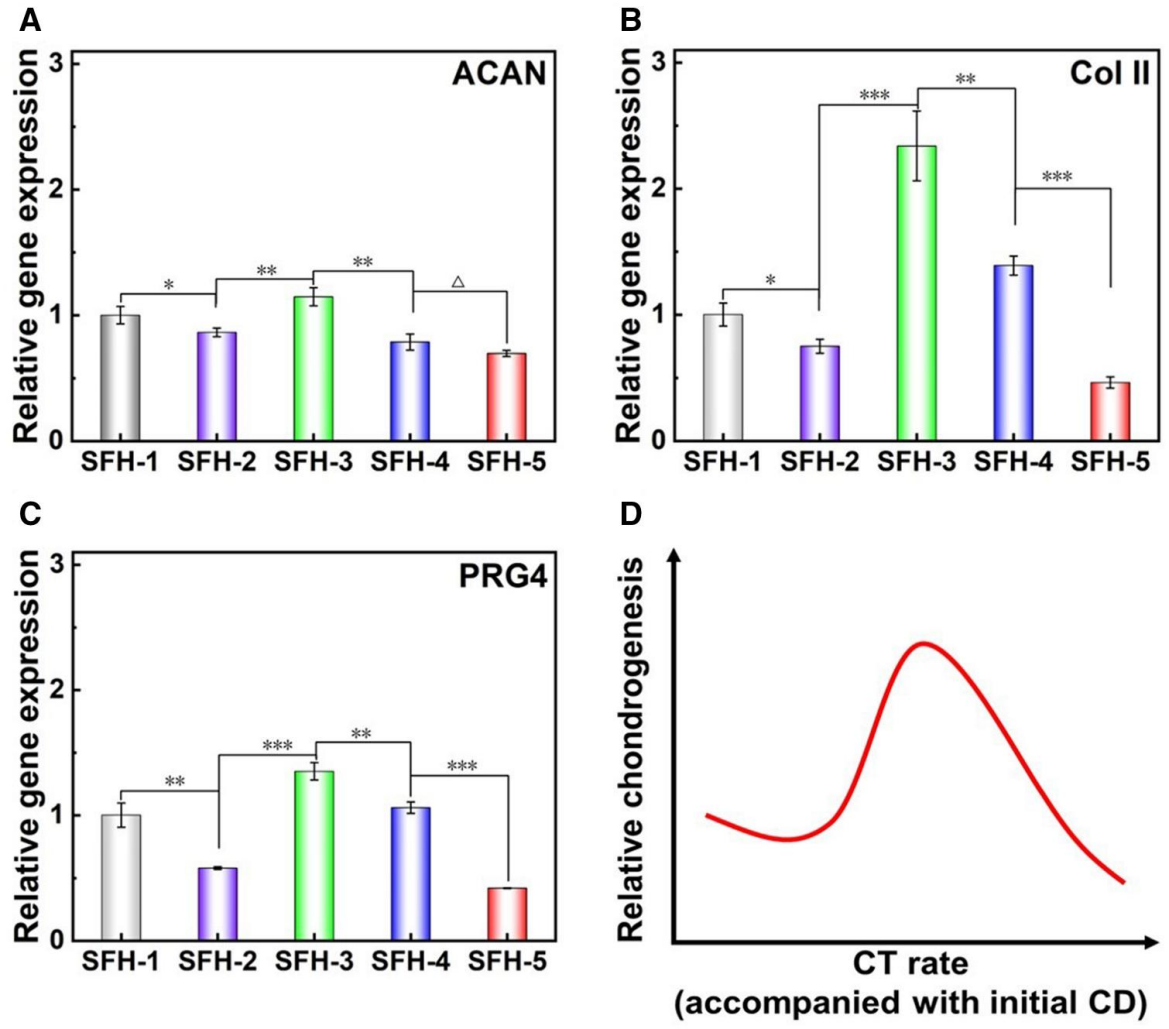
Chondrogenesis gene expression of encapsulated MSCs in SF hydrogels with different conformational transition rates after 7 days of culture. (**A**) Relative gene expression of ACAN, (**B**) relative gene expression of Col II, (**C**) relative gene expression of PRG4 and (**D**) schematic diagram of the influence of protein conformational transition rate (accompanied with different initial crosslinking densities) in SF hydrogel on the differentiation of encapsulated stem cells. CT, conformational transition; CD, crosslinking density. ‘Δ’: *P *>* *0.05; ‘*’: 0.01 < *P *<* *0.05; ‘**’: 0.001 < *P *<* *0.01; ‘***’: *P *<* *0.001. The *P* values of the one-way ANOVA test of the data in (**A**–**C**) are listed in supplementary Tables S6–S8.

It has been reported that the SFH showed great application potential in the field of cartilage tissue engineering [17, 34, 35]. So, this study chose this direction as a typical application scenario and carefully investigated the effects of protein conformational transition in SFHs on the chondrogenesis of encapsulated MSCs. The relative expression of chondrogenic characteristic genes of MSCs cultured for 7 days in the indicated SFHs is shown in Figure 7. Results illustrated that the highest expressions of ACAN, Col II and PRG4 are all presented in SFH-3 with medium conformational transition rate. So, SFH-3 shows an optimal induction property for stem cell chondrogenic differentiation. Referring to SFH-3, as the conformational transition process slowing down (SFH-2 and SFH-1) or speeding up (SFH-4 and SFH-5), the chondrogenic differentiation ability of the encapsulated stem cells decreased, as schematically summarized in Figure 7D. More specifically, the comprehensive chondrogenesis-inducing ability showed a following trend: SFH-3> SFH-4>SFH-1>SFH-2>SFH-5.

In the 3D cell culture environment, factors such as the pore size of the matrix, cell density, cell aggregation and dynamic mechanical stimulation could all have important roles for regulating the differentiation of stem cells. For example, it has been reported that dense cell aggregation was a key factor for the cartilage formation, as circular cell shape and high cell–cell contact were both conducive to cell chondrogenesis [80–82]. Therefore, a higher cell density within the matrix could be beneficial for the chondrogenic differentiation of stem cells [83]. In addition, relevant published works have also found that a relatively small pore-sized matrix could promote the aggregation of cells within the local matrix regions, thus providing a beneficial microenvironment for cell chondrogenesis [40, 84]. So, under the condition of satisfying normal cell survival, a relatively small pore size is beneficial to the chondrogenic differentiation of encapsulated stem cells. Moreover, some studies have also reported that appropriate matrix dynamic contraction (from large to small size) and dynamic stiffening (from low to high modulus) processes are both beneficial to cell chondrogenesis [38, 85].

During the initial 7 days, as the protein conformational transition occurred in the hydrogel, the pore size of the hydrogel gradually decreased (Figure 4), while the compressive mechanical properties gradually increased (Figure 5), presenting a dynamic process of stiffening and contraction. And these kinds of dynamic feature change rates were positively correlated with the mentioned conformational transition rate. In addition, the cell proliferation results showed that at day 7, SFH-3 and SFH-4 with a medium conformational transition process showed a much higher number of cells, while the hydrogels with a slower (SFH-2 and SFH-1) or faster (SFH-5) conformational transition process showed a much smaller number of cells (Figure 6A). As for SFH-3 or SFH-4, it is likely that the higher cell number, appropriate medium pore size, effective material contraction and stiffening properties have been combined together to obviously improve cell chondrogenesis compared to other groups. When comparing SFH-3 to SFH-4, the smaller pore size of SFH-3 could make the dynamical material cues more perceivable for cell aggregates, and the total cell number was also higher, which collectively led to a strongest chondrogenic differentiation on SFH-3. As for SFH-2 and SFH-1, the conformational transition process slows down (Figure 5). There is also a significant reduction in cell number in SFH-2 and SFH-1 (Figure 6). The weak dynamic contraction and stiffening process and the lower cell number during the initial 7 days collectively lead to an obviously decreased chondrogenesis induction ability. In addition, the conformational transitions of SFH-1 and SFH-2 are notably slow. Consequently, the increased chemical crosslinking density in SFH-1 leads to a smaller pore size relative to SFH-2 (Figure 4). This characteristic pore size in SFH-1 may further promote a higher local cell density and enhance cell aggregation compared with SFH-2. That is probably why the chondrogenesis induction ability of SFH-1 was higher than that of SFH-2. As for SFH-5, although the dynamic material cues changed fastest (Figure 5), its overly large pore size probably could not induce effective cell aggregates and make the dynamic material cues less perceivable at the initial stage. At the later stage of 1–7 days, the pores have sharply collapsed (Figure 4), which become unfriendly for cell growth and proliferation. Combined with these unique features, it finally led to a weakest chondrogenesis induction ability of SFH-5.

**Figure 8.**
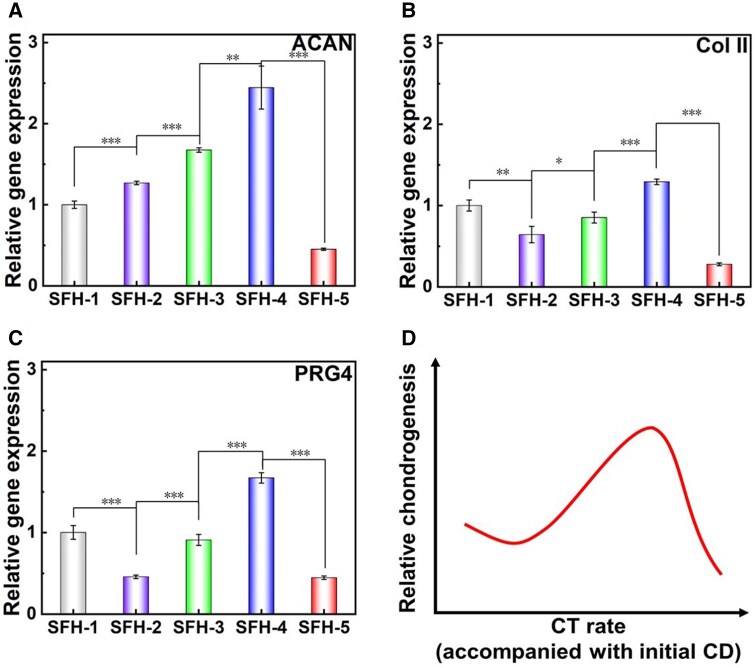
Chondrogenesis gene expression of encapsulated MSCs in SF hydrogels with different conformational transition rates after 14 days of culture. (**A**) Relative gene expression of ACAN, (**B**) relative gene expression of Col II, (**C**) relative gene expression of PRG4 and (**D**) schematic diagram of the influence of protein conformational transition rate (accompanied with different initial crosslinking densities) in SF hydrogel on the differentiation of encapsulated stem cells. CT, conformational transition; CD, crosslinking density. ‘*’: 0.01 < *P *<* *0.05; ‘**’: 0.001 < *P *<* *0.01; ‘***’: *P *<* *0.001. The *P* values of the one-way ANOVA test of the data in (**A**–**C**) are listed in Supplementary Tables S9–S11.

When further culturing the cells to 14 days, the relative chondrogenic characteristic gene expression is shown in Figure 8A–C. Compared with the trend of 7 days (Figure 7D), the difference was that the group exhibiting the highest chondrogenesis has changed from SFH-3 to SFH-4. More specifically, the comprehensive chondrogenesis inducing ability showed a following trend: SFH-4>SFH-3>SFH-1>SFH-2>SFH-5, as schematically summarized and illustrated in Figure 8D.

During the culture period from 7 to 14 days, as the protein conformational transition developed, the pore size of the hydrogels progressively decreased (Figure 4), and the compressive mechanical properties further significantly increased (Figure 5), presenting a more obvious dynamic stiffening and contraction process. These indicated dynamic material feature change rates were still positively correlated with the conformational transition of the related hydrogels. During this period, the dynamical material features might become the main influencing factor for regulating cell chondrogenesis as they changed much faster when compared to the initial 7 days. When comparing SFH-3 and SFH-4, SFH-4 presented a more significant dynamic stiffening and contraction process (Figure 5) during this period. In addition, the fast pore size decreases in SFH-4 (Figure 4) further enhanced local cell density and effective cell aggregation. These two factors could be attributed to the highest chondrogenesis on SFH-4. As for SFH-5, although the conformational transition rate is the fastest, its pore structure collapse and overly small pore size obviously restricted cell growth and nutrient exchange, thus leading to a lowest chondrogenic differentiation induction ability. As the conformational transition process slows down (SFH-2 and SFH-1), it is likely that the weak dynamic contraction and stiffening cues lead to a decreased chondrogenesis induction ability compared with SFH-4 and SFH-3. In addition, because the conformational transition of SFH-2 and SFH-1 were both very slow, the pore size of SFH-1 was constantly smaller than that of SFH-2, which may induce more effective cell aggregation. That is probably why the chondrogenesis induction ability of SFH-1 was still higher than that of SFH-2.

Comprehensively, an appropriate rate of conformational transition and initial crosslinking density of SFH is beneficial for promoting the chondrogenic differentiation of encapsulated stem cells. When the conformational transition becomes faster or slower, the corresponding chondrogenic differentiation induction ability decreases to some extent. The aforementioned results were probably determined by the initial hydrogel crosslinking density accompanied with the following dynamical material feature changes induced by the protein conformational transition process.

## Conclusions

In this study, based on the strategy of controlling hydrogel crosslinking density, a simple and effective method was successfully developed to regulate the protein conformational transition process of SFHs. The lower the crosslinking density, the faster the conformational transition process. As the conformational transition occurs, the transparency of the hydrogel gradually decreased, the microscopic network and pore structure gradually contracted, and the compressive strength and modulus gradually increased, comprehensively showing a dynamic contraction and stiffening process. These dynamic material characteristics’ change rates were positively correlated with the conformational transition rate of related hydrogels. Moreover, this research further revealed that the mentioned conformational transition process accompanied with related crosslinking density could effectively regulate the proliferation and chondrogenesis of encapsulated stem cells. It was ultimately illustrated that SFHs with moderate conformational transition process could significantly enhance the proliferation and chondrogenic differentiation of MSCs, while too fast or too slow conformational transition processes could slow down the mentioned cell behaviors. The revealed impacts on cell behaviors were probably attributed to the effects of the dynamical protein conformational transition process accompanied with the initial hydrogel crosslinking density. This research is hopefully to provide effective guidance for the development and application of SFH scaffolds in the field of cartilage repair. It could also provide new ideas and references for the efficient design, development and application of SF and other protein-based biomaterials.

## Supplementary Material

rbaf019_Supplementary_Data
