# Supplementary material for: Effects of protein conformational transition accompanied with crosslinking density cues in silk fibroin hydrogels on the proliferation and chondrogenesis of encapsulated stem cells
Source: Regen Biomater. 2025 Mar 20;12:rbaf019. doi: 10.1093/rb/rbaf019 (PMC12033033; doi:10.1093/rb/rbaf019)
Supplement: rbaf019_Supplementary_Data [file rbaf019_supplementary_data.zip › Supporting Information-20250201.docx]

Supporting Information for

**Effects of protein conformational transition accompanied with crosslinking density cues in silk fibroin hydrogels on the proliferation and chondrogenesis of encapsulated stem cells**

Guolong Cai ^a^, Weikun Zhao ^a^, Tianhao Zhu ^a^, Ana L. Oliveira ^b^, Xiang Yao ^a,*^, Yaopeng Zhang ^a,*^

^a^ State Key Laboratory of Advanced Fiber Materials, Shanghai Engineering Research Center of Nano-Biomaterials and Regenerative Medicine, College of Materials Science and Engineering, Donghua University, Shanghai 201620, People’s Republic of China

^b^ Universidade Católica Portuguesa, CBQF - Centro de Biotecnologia e Química Fina – Laboratório Associado, Escola Superior de Biotecnologia, Rua Diogo Botelho 1327, 4169-005 Porto, Portugal

*Correspondence address. E-mail: yaoxiang@dhu.edu.cn (X.Y.); zyp@dhu.edu.cn (Y.Z.)


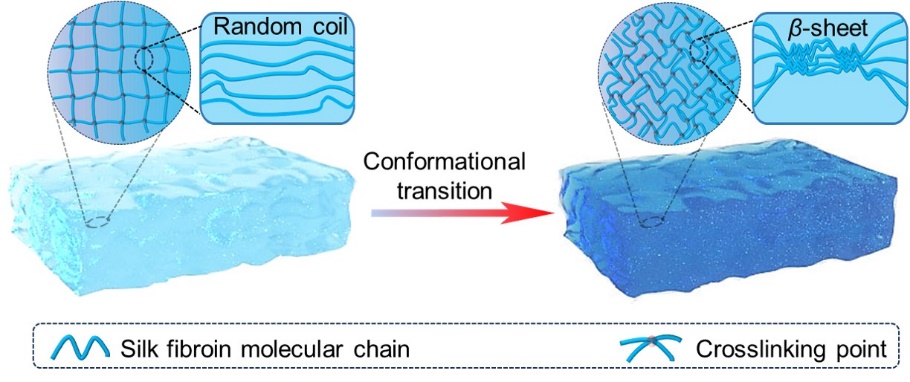


**Figure S1** Schematic illustration of the protein conformational transition in the corresponding chemical crosslinked SF hydrogel.

**Table S1** Precursor solution formulations of the indicated SF hydrogels

| Sample code | *V*_(SF)_/mL | | *V*_(HRP)_/μL | *V*_(H2O2)_/μL | |
| --- | --- | --- | --- | --- | --- |
| SFH-1 | 2 | 100 | | | 52.5 |
| SFH-2 | 2 | 80 | | | 42.0 |
| SFH-3 | 2 | 60 | | | 31.5 |
| SFH-4 | 2 | 50 | | | 26.3 |
| SFH-5 | 2 | 40 | | | 21.0 |

Notes：The concentration of SF solution is 5 wt%; The initial concentration of HRP solution is 1000 U/mL；the initial concentration of H_2_O_2_ is 490 mM.

**Table S2** RT-PCR primer sequences of the chondrogenic characteristic genes.

| **Gene name** |  | **Sequences** |
| --- | --- | --- |
| Col Ⅱ | Forward primer | 5' GGCGAGTCTTGCGTCTAC 3' |
|  | Reverse primer | 5' GTGCTTCTTCTCCTTGCTCTT 3' |
| ACAN | Forward primer | 5' ATCTATCGCTGTGAAGTGATG 3' |
|  | Reverse primer | 5' CTCGGTCAAAGTCCAGTGT 3' |
| PRG4 | Forward primer | 5' GTATTCCCTCTCCCATTGAC 3' |
|  | Reverse primer | 5' GATACCCAGCATCCATTACAT 3' |
| GAPDH | Forward primer | 5' TGTTCCTACCCCCAATGTAT 3' |
|  | Reverse primer | 5' TTCACCACCTCCTTGATGTC 3' |


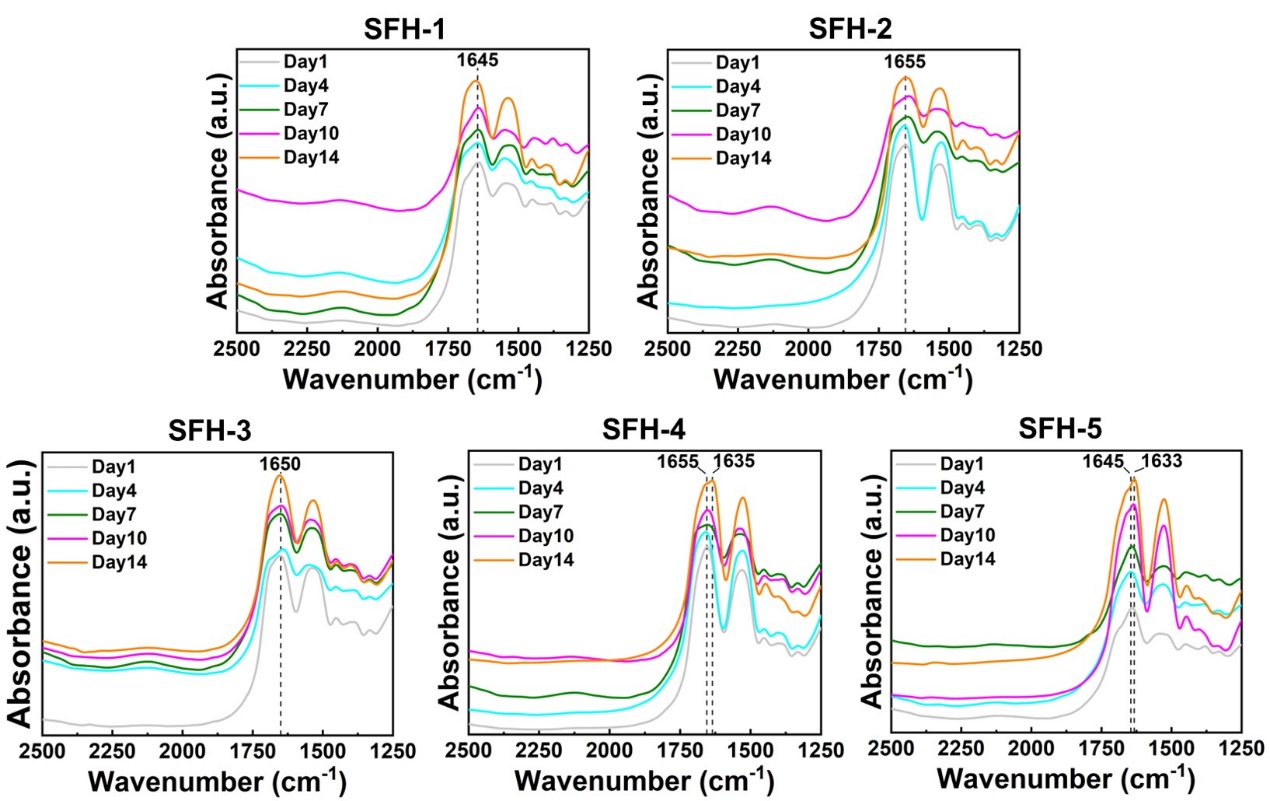


**Figure S2** FTIR curves of the SF hydrogels after indicated incubation times in the simulated cell culture environment.


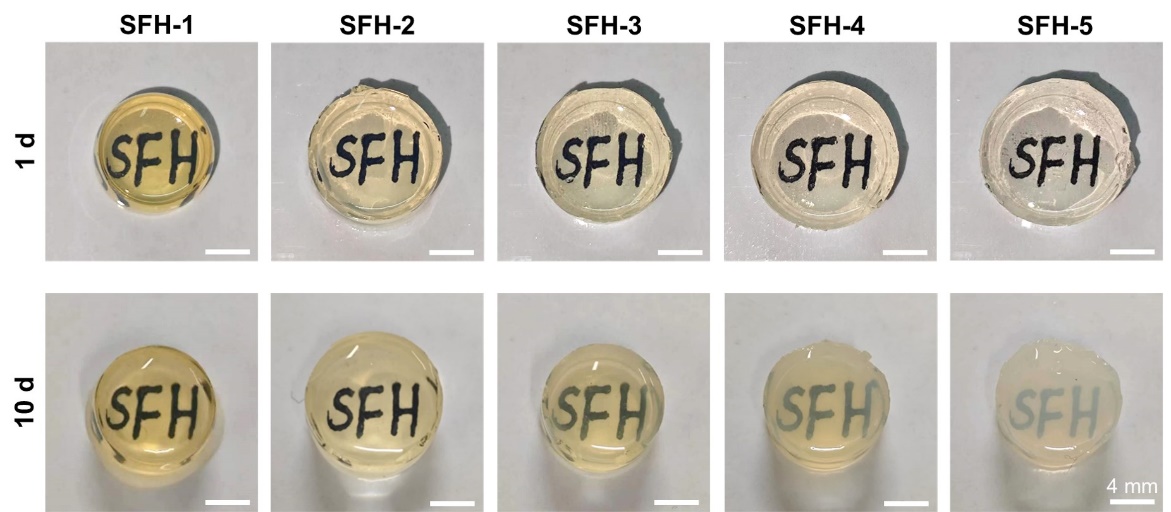


**Figure S3** Gross views of the corresponding hydrogels after 1 day and 10 days of incubation.


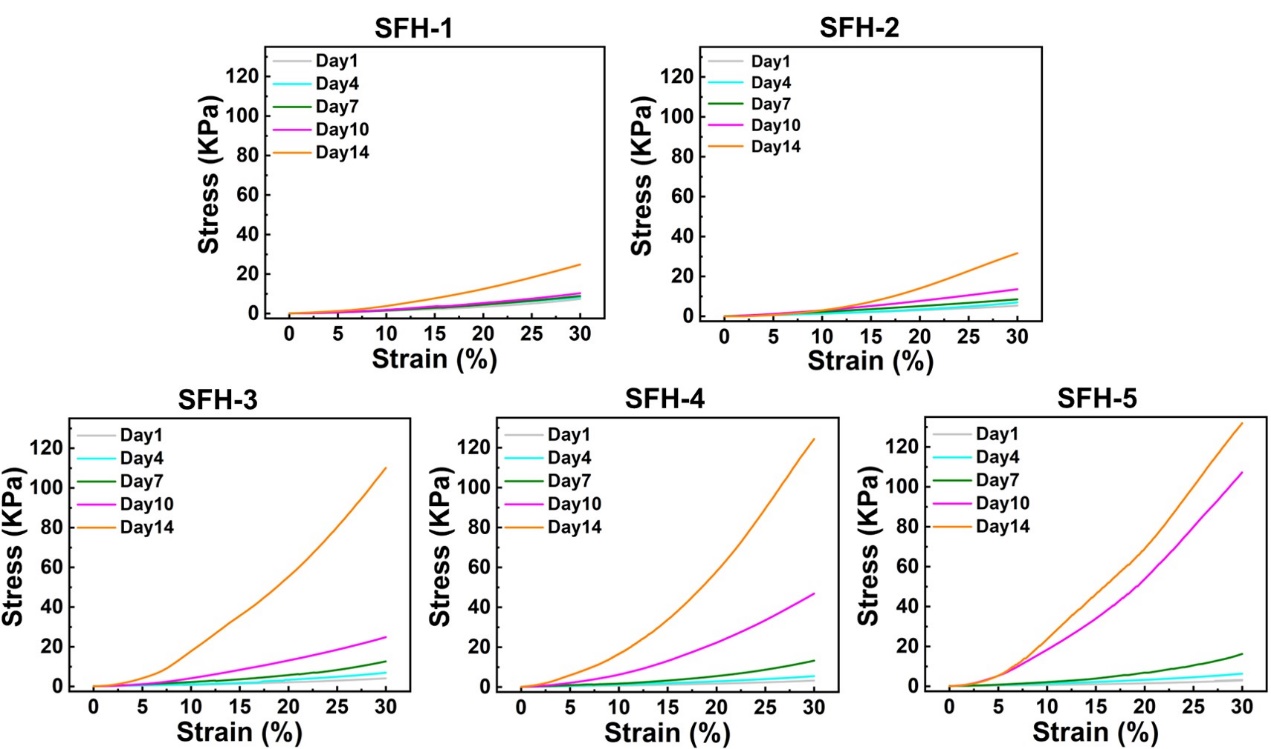


**Figure S4** Stress-strain curves of the SF hydrogels after indicated incubation times in the simulated cell culture environment.

**Table S3** The *p* value of the One-way ANOVA test for luminescence intensity detected after cells cultured in different SF hydrogels for 1 d in Fig. 6A

| Luminescence intensity | SFH-1 | SFH-2 | SFH-3 | SFH-4 | SFH-5 |
| --- | --- | --- | --- | --- | --- |
| SFH-1 | / | *** | *** | *** | *** |
|  |  | 1.29×10^-12^ | 1.02×10^-13^ | 1.12×10^-17^ | 3.00×10^-18^ |
| SFH-2 | *** | / | *** | *** | *** |
|  | 1.29×10^-12^ |  | 1.53×10^-8^ | 5.22×10^-12^ | 2.39×10^-13^ |
| SFH-3 | *** | *** | / | * | *** |
|  | 1.02×10^-13^ | 1.53×10^-8^ |  | 1.01×10^-2^ | 7.80×10^-7^ |
| SFH-4 | *** | *** | * | / | *** |
|  | 1.12×10^-17^ | 5.22×10^-12^ | 1.01×10^-2^ |  | 1.32×10^-6^ |
| SFH-5 | *** | *** | *** | *** | / |
|  | 3.00×10^-18^ | 2.39×10^-13^ | 7.80×10^-7^ | 1.32×10^-6^ |  |

“*”: 0.01 < *p* < 0.05, significant difference; “***”: *p* < 0.001, significant difference

**Table S4** The *p* value of the One-way ANOVA test for luminescence intensity detected after cells cultured in different SF hydrogels for 4 d in Fig. 6A

| Luminescence intensity | SFH-1 | SFH-2 | SFH-3 | SFH-4 | SFH-5 |
| --- | --- | --- | --- | --- | --- |
| SFH-1 | / | *** | *** | *** | *** |
|  |  | 1.80×10^-15^ | 7.39×10^-17^ | 3.56×10^-20^ | 4.88×10^-16^ |
| SFH-2 | *** | / | *** | *** | *** |
|  | 1.80×10^-15^ |  | 1.65×10^-13^ | 9.02×10^-17^ | 3.04×10^-14^ |
| SFH-3 | *** | *** | / | *** | *** |
|  | 7.39×10^-17^ | 1.65×10^-13^ |  | 3.98×10^-6^ | 1.09×10^-4^ |
| SFH-4 | *** | *** | *** | / | Δ |
|  | 3.56×10^-20^ | 9.02×10^-17^ | 3.98×10^-6^ |  | 0.470 |
| SFH-5 | *** | *** | *** | Δ | / |
|  | 4.88×10^-16^ | 3.04×10^-14^ | 1.09×10^-4^ | 0.470 |  |

“**Δ**”: *p* > 0.05, no significant difference; “***”: *p* < 0.001, significant difference

**Table S5** The *p* value of the One-way ANOVA test for luminescence intensity detected after cells cultured in different SF hydrogels for 7 d in Fig. 6A

| Luminescence intensity | SFH-1 | SFH-2 | SFH-3 | SFH-4 | SFH-5 |
| --- | --- | --- | --- | --- | --- |
| SFH-1 | / | *** | *** | *** | *** |
|  |  | 2.18×10^-4^ | 1.87×10^-8^ | 8.14×10^-14^ | 3.10×10^-10^ |
| SFH-2 | *** | / | *** | *** | *** |
|  | 2.18×10^-4^ |  | 2.06×10^-8^ | 9.14×10^-11^ | 2.02×10^-6^ |
| SFH-3 | *** | *** | / | * | *** |
|  | 1.87×10^-8^ | 2.06×10^-8^ |  | 2.95×10^-2^ | 3.06×10^-6^ |
| SFH-4 | *** | *** | * | / | *** |
|  | 8.14×10^-14^ | 9.14×10^-11^ | 2.95×10^-2^ |  | 3.27×10^-9^ |
| SFH-5 | *** | *** | *** | *** | / |
|  | 3.10×10^-10^ | 2.02×10^-6^ | 3.06×10^-6^ | 3.27×10^-9^ |  |

“*”: 0.01 < *p* < 0.05, significant difference; “***”: *p* < 0.001, significant difference

**Table S6** The *p* value of the One-way ANOVA test for relative ACAN expression detected after cells cultured in different SF hydrogels for 7 d in Fig. 7A

| relative ACAN expression | SFH-1 | SFH-2 | SFH-3 | SFH-4 | SFH-5 |
| --- | --- | --- | --- | --- | --- |
| SFH-1 | / | * | Δ | * | ** |
|  |  | 3.77×10^-2^ | 6.33×10^-2^ | 1.70×10^-2^ | 2.00×10^-3^ |
| SFH-2 | * | / | ** | Δ | ** |
|  | 3.77×10^-2^ |  | 3.51×10^-3^ | 0.141 | 2.51×10^-3^ |
| SFH-3 | Δ | ** | / | ** | *** |
|  | 6.33×10^-2^ | 3.51×10^-3^ |  | 2.87×10^-3^ | 4.97×10^-4^ |
| SFH-4 | * | Δ | ** | / | Δ |
|  | 1.70×10^-2^ | 0.141 | 2.87×10^-3^ |  | 8.30×10^-2^ |
| SFH-5 | ** | ** | *** | Δ | / |
|  | 2.00×10^-3^ | 2.51×10^-3^ | 4.97×10^-4^ | 8.30×10^-2^ |  |

“**Δ**”: *p* > 0.05, no significant difference; “*”: 0.01 < *p* < 0.05, significant difference; “**”:0.001 < *p* < 0.01, significant difference; “***”: *p* < 0.001, significant difference

**Table S7** The *p* value of the One-way ANOVA test for relative Col II expression detected after cells cultured in different SF hydrogels for 7 d in Fig. 7B

| relative Col II expression | SFH-1 | SFH-2 | SFH-3 | SFH-4 | SFH-5 |
| --- | --- | --- | --- | --- | --- |
| SFH-1 | / | * | ** | ** | *** |
|  |  | 1.51×10^-2^ | 1.34×10^-3^ | 4.77×10^-3^ | 7.85×10^-4^ |
| SFH-2 | * | / | *** | *** | ** |
|  | 1.51×10^-2^ |  | 6.10×10^-4^ | 2.84×10^-4^ | 2.13×10^-3^ |
| SFH-3 | ** | *** | / | ** | *** |
|  | 1.34×10^-3^ | 6.10×10^-4^ |  | 4.52×10^-3^ | 3.11×10^-4^ |
| SFH-4 | ** | *** | ** | / | *** |
|  | 4.77×10^-3^ | 2.84×10^-4^ | 4.52×10^-3^ |  | 5.13×10^-5^ |
| SFH-5 | *** | ** | *** | *** | / |
|  | 7.85×10^-4^ | 2.13×10^-3^ | 3.11×10^-4^ | 5.13×10^-5^ |  |

“*”: 0.01 < *p* < 0.05, significant difference; “**”:0.001 < *p* < 0.01, significant difference; “***”: *p* < 0.001, significant difference

**Table S8** The *p* value of the One-way ANOVA test for relative PRG4 expression detected after cells cultured in different SF hydrogels for 7 d in Fig. 7C

| relative PRG4 expression | SFH-1 | SFH-2 | SFH-3 | SFH-4 | SFH-5 |
| --- | --- | --- | --- | --- | --- |
| SFH-1 | / | ** | ** | Δ | *** |
|  |  | 1.63×10^-3^ | 7.01×10^-3^ | 0.390 | 4.69×10^-4^ |
| SFH-2 | ** | / | *** | *** | *** |
|  | 1.63×10^-3^ |  | 4.41×10^-5^ | 5.53×10^-5^ | 1.40×10^-5^ |
| SFH-3 | ** | *** | / | ** | *** |
|  | 7.01×10^-3^ | 4.41×10^-5^ |  | 3.70×10^-3^ | 2.00×10^-5^ |
| SFH-4 | Δ | *** | ** | / | *** |
|  | 0.390 | 5.53×10^-5^ | 3.70×10^-3^ |  | 1.61×10^-5^ |
| SFH-5 | *** | *** | *** | *** | / |
|  | 4.69×10^-4^ | 1.40×10^-5^ | 2.00×10^-5^ | 1.61×10^-5^ |  |

“**Δ**”: *p* > 0.05, no significant difference; “**”:0.001 < *p* < 0.01, significant difference; “***”: *p* < 0.001, significant difference

**Table S9** The *p* value of the One-way ANOVA test for relative ACAN expression detected after cells cultured in different SF hydrogels for 14 d in Fig. 8A

| relative ACAN expression | SFH-1 | SFH-2 | SFH-3 | SFH-4 | SFH-5 |
| --- | --- | --- | --- | --- | --- |
| SFH-1 | / | *** | *** | *** | *** |
|  |  | 7.88×10^-4^ | 2.51×10^-5^ | 7.32×10^-4^ | 3.87×10^-5^ |
| SFH-2 | *** | / | *** | ** | *** |
|  | 7.88×10^-4^ |  | 3.34×10^-5^ | 1.54×10^-3^ | 6.85×10^-7^ |
| SFH-3 | *** | *** | / | ** | *** |
|  | 2.51×10^-5^ | 3.34×10^-5^ |  | 7.34×10^-3^ | 2.45×10^-7^ |
| SFH-4 | *** | ** | ** | / | *** |
|  | 7.32×10^-4^ | 1.54×10^-3^ | 7.34×10^-3^ |  | 1.98×10^-4^ |
| SFH-5 | *** | *** | *** | *** | / |
|  | 3.87×10^-5^ | 6.85×10^-7^ | 2.45×10^-7^ | 1.98×10^-4^ |  |

“**”:0.001 < *p* < 0.01, significant difference; “***”: *p* < 0.001, significant difference

**Table S10** The *p* value of the One-way ANOVA test for relative Col II expression detected after cells cultured in different SF hydrogels for 14 d in Fig. 8B

| relative Col II expression | SFH-1 | SFH-2 | SFH-3 | SFH-4 | SFH-5 |
| --- | --- | --- | --- | --- | --- |
| SFH-1 | / | ** | Δ | ** | *** |
|  |  | 7.03×10^-3^ | 5.56×10^-2^ | 2.72×10^-3^ | 5.94×10^-5^ |
| SFH-2 | ** | / | * | *** | ** |
|  | 7.03×10^-3^ |  | 4.05×10^-2^ | 4.54×10^-4^ | 3.51×10^-3^ |
| SFH-3 | Δ | * | / | *** | *** |
|  | 5.56×10^-2^ | 4.05×10^-2^ |  | 5.53×10^-4^ | 1.43×10^-4^ |
| SFH-4 | ** | *** | *** | / | *** |
|  | 2.72×10^-3^ | 4.54×10^-4^ | 5.53×10^-4^ |  | 1.37×10^-6^ |
| SFH-5 | *** | ** | *** | *** | / |
|  | 5.94×10^-5^ | 3.51×10^-3^ | 1.43×10^-4^ | 1.37×10^-6^ |  |

“**Δ**”: *p* > 0.05, no significant difference; “*”: 0.01 < *p* < 0.05, significant difference; “**”:0.001 < *p* < 0.01, significant difference; “***”: *p* < 0.001, significant difference

**Table S11** The *p* value of the One-way ANOVA test for relative PRG4 expression detected after cells cultured in different SF hydrogels for 14 d in Fig. 8C

| relative PRG4 expression | SFH-1 | SFH-2 | SFH-3 | SFH-4 | SFH-5 |
| --- | --- | --- | --- | --- | --- |
| SFH-1 | / | *** | Δ | *** | *** |
|  |  | 4.14×10^-4^ | 0.214 | 3.93×10^-4^ | 3.84×10^-4^ |
| SFH-2 | *** | / | *** | *** | Δ |
|  | 4.14×10^-4^ |  | 3.87×10^-4^ | 6.11×10^-6^ | 0.556 |
| SFH-3 | Δ | *** | / | *** | *** |
|  | 0.214 | 3.87×10^-4^ |  | 1.43×10^-4^ | 3.54×10^-4^ |
| SFH-4 | *** | *** | *** | / | *** |
|  | 3.93×10^-4^ | 6.11×10^-6^ | 1.43×10^-4^ |  | 5.93×10^-6^ |
| SFH-5 | *** | Δ | *** | *** | / |
|  | 3.84×10^-4^ | 0.556 | 3.54×10^-4^ | 5.93×10^-6^ |  |

“**Δ**”: *p* > 0.05, no significant difference; “***”: *p* < 0.001, significant difference
